# Single-cell transcriptomic profiling of human fetal neural stem cells isolated from the subventricular zone

**DOI:** 10.3389/fcell.2026.1740851

**Published:** 2026-03-10

**Authors:** Domenico Alessandro Silvestris, Annunziata De Luisi, Diletta Lucia Capobianco, Francesco Di Palma, Daniela Profico, Maurizio Gelati, Andrea Gerbino, Angelo Luigi Vescovi, Maria Svelto, Ernesto Picardi, Graziano Pesole, Francesco Pisani, Anna Maria D'Erchia

**Affiliations:** 1 Department of Biosciences, Biotechnologies and Environment, University of Bari “Aldo Moro”, Bari, Italy; 2 ProPharma LLC, Leiden, Netherlands; 3 Fondazione IRCSS Istituto Neurologico “C. Besta”, Milan, Italy; 4 Faculty of Medicine, Link Campus University, Rome, Italy; 5 Institute of Biomembranes, Bioenergetics and Molecular Biotechnology, National Research Council, Bari, Italy

**Keywords:** neural stem cells, neurodegenerative diseases, regenerative medicine, ScRNA-seq, transcriptomics

## Abstract

Neural stem cells (NSCs) are self-renewing, multipotent cells capable of differentiating into neurons, astrocytes, and oligodendrocytes. NSCs reside in specific brain niches: the ventricular zone (VZ), the subventricular zone (SVZ) and the subgranular zone of the dentate gyrus (SGZ). Each of these niches orchestrates finely tuned neurogenesis and gliogenesis in both the developing neocortex and the adult brain. The exact cellular composition of human NSCs (hNSCs) from the SVZ (SVZ-hNSCs) of the developing human neocortex is not yet fully understood and remains elusive. This represents a major obstacle to understanding how human neurogenesis works and limits the potential of fetal derived hNSCs in regenerative medicine applications. To address this, we performed single-cell transcriptome analysis on hNSCs, isolated from the SVZ of fetal brains of different donors, resulting from spontaneous miscarriages, and subjected to different culture passages. Our analysis revealed, in each sample, the high expression of the canonical stemness markers. Among the identified sub-populations, we observed a neural progenitor cell cluster, also expressing typical markers of the quiescent state. The remaining clusters diverged from this population along two main trajectories: one oriented toward the glial lineage and another with a more neuronal identity. Our analysis further revealed that extended *in vitro* culture induces a progressive transcriptional shift, characterized by the activation of differentiation programs, providing insights into the temporal dynamics of hNSCs identity. Nevertheless, despite this gradual transcriptional shift, late passage cultured hNSCs retained their stemness, as the strongly stem-like cluster persisted with a high number of cells. Overall, our findings provide a deeper characterization of hNSCs isolated from the fetal brain and demonstrate their long-term stability and safety in regenerative medicine, as they preserve their stem-like identity even after prolonged *in vitro* expansion.

## Introduction

During the early stages of mammalian fetal brain development, neuroepithelial cells generate apical radial glial cells (aRGs) that reside in the ventricular zone (VZ), lining the cerebral ventricles (Götz and Huttner, 2005; Yamashita, 2013; Taverna et al., 2014). Subsequently, a second germinal layer, the subventricular zone (SVZ), emerges. The SVZ is mainly populated by basal progenitors, including basal intermediate progenitors and basal radial glial cells (bRGs), although its cellular composition changes dynamically during fetal development (Hansen et al., 2010; Fietz and Huttner, 2011; Reillo et al., 2011; Liu et al., 2023). aRGs and bRGs—collectively referred to as neural stem cells (NSCs)—orchestrate neurogenesis, differentiating first into neurons (neurogenesis) and later into astrocytes and oligodendrocytes (gliogenesis) (Vieira et al., 2018). Among the cell types populating the SVZ, bRGs are considered key contributors to the evolutionary expansion of the mammalian neocortex (Borrell and Reillo, 2012; Florio and Huttner, 2014; Kalebic et al., 2019).

In adult mammals, neurogenesis involves the proliferation and fate specification of adult neural progenitor cells (NPCs), their migration, site specific differentiation and functional integration in existing neural circuits, in response to specific stimuli (Ming and Song, 2005).

Due to their capability to self-renew and differentiate into multiple neural lineages, human NSCs (hNSCs) are emerging as promising tools for regenerative medicine in neurodegenerative diseases (Rahimi Darehbagh et al., 2024). In particular, the human fetal SVZ represents a valuable source for the isolation, expansion, and stabilization of good manufacturing practice (GMP)-grade NSCs for potential use in cell-based therapies (Profico et al., 2022). Recently, in an early-phase clinical trial, cohorts of patients with progressive multiple sclerosis (MS) and amyotrophic lateral sclerosis (ALS) were treated with GMP-grade hNSCs isolated from the fetal SVZ at gestational weeks (GW) 15–16, delivered via intracerebroventricular injection (MS) or intraspinal transplantation (ASL). The treatments were well tolerated, with no adverse events and stable clinical and laboratory outcomes (Mazzini et al., 2019; Leone et al., 2023).

Although it is known that the VZ and SVZ of the human fetal brain at GW 14–17 contain high levels of aRGs, bRGs, and intermediate progenitor cells (Eşiyok and Heide, 2023), the exact cellular and molecular composition of hNSCs isolated and cultured from GW 15–16 tissue remains largely unknown.

Recently, single-cell RNA sequencing (scRNA-seq) has revolutionized the fields of biology and medicine, by enabling the transcriptomic profiling of individual cells. This technology allows for the characterization of RNA heterogeneity and complexity within individual cells, and reveals the diversity of cell types and their functions within tissues (Tang et al., 2011). Thus, scRNA-seq represents a powerful tool to investigate the molecular features of hNSCs, providing new insights into their cellular composition and heterogeneity. Here, we used scRNA-seq to perform single cell transcriptomic profiling of hNSCs isolated from the SVZ of fetal brains from different donors, at GW15-16, resulting from spontaneous miscarriage. After isolation, hNSCs were expanded for several culture passages and analysed at both early and late passages, to assess whether culture duration could affect stemness. Our analysis revealed, in each sample, the high expression of the canonical stemness markers, even at late passages. Among the identified sub-populations, we observed a neural progenitor cell cluster, also expressing typical markers of the quiescent state, as AQP4 and APOE. The remaining clusters diverged from this population along two main trajectories: one oriented toward the glial lineage, and another with a more neuronal identity. Finally, our data revealed that prolonged *in vitro* culture induces a progressive transcriptional shift, marked by the activation of differentiation programs, providing insights into the temporal dynamics of hNSC identity.

## Materials and methods

### Human neural stem cell isolation and culture

Human NSCs were isolated from the brain of fetal human donors following spontaneous miscarriage at 15–16 weeks of gestation (GW). All samples derived from fetuses with healthy karyotypes, performed both on skin and directly on hNSCs, confirmed with SNP arrays. Furthermore, the fetuses had no phenotypic signs of abnormalities and serological screening was negative for infectious diseases tested in accordance with the regulations in force at the time of sampling. The hNSCs were also tested *in vivo* for non-tumorigenicity, with transplants in nude mice (Profico et al., 2022). Four different samples were processed. The isolation procedure consisted of two main steps: (i) primary culture and (ii) intermediate product preparation and cryopreservation, as previously described (Profico et al., 2022; Leone et al., 2023; Capobianco et al., 2024). For the primary culture, brain tissue was immediately transferred, under strict sterile conditions, to the GMP facility, maintained in a controlled environment and washed in PBS (Dulbecco’s PBS 1X, Carlo Erba Reagent), supplemented with 50 μg/mL gentamicin. The tissue was mechanically dissociated to obtain a monocellular suspension. Cells were seeded at a density of 104 cells/cm^2^ in a chemically defined culture medium supplemented with EGF 20 ng/mL and bFGF 10 ng/mL. Cultures were maintained in a humidified incubator at 37 °C, 5% O_2_, and 5% CO_2_ allowing cells to proliferate as free-floating clusters (called neurospheres), according to the standard ‘neurosphere assay technique’. Approximately 7–10 days after primary cell seeding, neurospheres were collected, centrifuged, and the resulting cell pellet mechanically dissociated by gentle pipetting. Cells were counted using a Burker chamber and evaluated for viability (95%–98%). Cells were subcultured every 7–12 days (passages, P) and cryopreserved in a culture medium with 10% dimethylsulfoxide, until thawing for further expansion.

### Single cell RNA-seq library preparation and sequencing

Approximately 7–10 days before scRNA-seq library preparation, neurospheres, at different passages, were thawed and allowed to grow in hNSC-medium at 37 °C, 5% O_2_, and 5% CO_2_, until processed. Neurospheres were collected, resuspended in PBS and mechanically dissociated into single cells, by pipetting. Then, cells were centrifuged at 200 rcf for 10 min and resuspended in 1 mL of 1X PBS containing 0.04% BSA, by pipetting 10–15 times, using a wide-bore pipette tip. This passage was repeated twice. Cell counting and viability were determined by mixing 10 μL of cell suspension with an equal volume of 0.4% trypan blue and transferring 10 μL of the mixture into a chamber of the TC20 Automated Cell Counter (Biorad). Only samples with >90% viability were further processed and resuspended in 1X PBS containing 0.04% BSA, in order to obtain a concentration of 800–1,500 cells/μL. Single cells were processed using the Chromium Single Cell 3′ Reagent Kit (v3.1) with the 10X Chromium Controller platform (10X Genomics), following the manufacturer’s protocol, expecting a target cell recovery of 5,000 cells. The resulting single cell libraries were evaluated for fragment size distribution, using the High Sensitivity DNA assay chip on the Bioanalyzer instrument (Agilent) and quantified with Qubit 1X dsDNA High Sensitivity assay kit (Thermo Fisher). Libraries were sequenced on the Illumina NovaSeq 6000 platform, available at the Italian Infrastructure for Omics and Bioinformatics ELIXIR-IT in Bari (Italy), generating approximately 20,000 paired reads per cell, made up as follows: read 1:28 cycles; read 2:120 cycles.

### Single cell RNA-seq data analysis

CellRanger 9.0.1 software (10X Genomics) was used to perform demultiplexing of the input files, alignment to the human reference genome (GRCh38) and transcriptome (GENCODE primary v48), using STAR software and UMI quantification to produce a cell-by-gene matrix for all samples, which were subsequently aggregated (without normalization). Single-cell UMI count matrices (Cell Ranger “filtered_feature_bc_matrix”) were loaded into Seurat (v5) (Hao et al., 2024) and samples annotated as NSC_P8, NSC_P16, NSC_P18, NSC_P22. Cells passed quality control (QC) if they expressed more than 200 genes (indication of low viability) and fewer than 6,000 genes (indication of doublets), with additional thresholds of percent.mt < 20, nFeature_RNA > 500 and *MALAT1* fraction < 5. Additional QC metrics included percent.ribo, percent.hist, *NEAT1/APOE/XIST* fractions, and log10GenesPerUMI. Cell-cycle scoring employed the 2019 S and G2/M lists (UpdateSymbolList), with cc.Difference = S.Score − G2M.Score. Each sample was independently normalized with Seurat’s standard method regressing for cc.Difference, percent.hist and percent.mt. Principal Component Analysis (PCA) was performed on the RNA assay, and batch/intersample effects were mitigated using Harmony “soft” integration (Korsunsky et al., 2019) on the PCA reduction. Dimensionality reductions with Uniform Manifold Approximation and Projection (UMAP) and t-distributed Stochastic Neighbor Embedding (t-SNE) were computed from Harmony embeddings (dims 1–30; Seurat defaults). A Shared-Nearest-Neighbor (SNN) graph was built on Harmony (dims 1–30), and clustering was performed using FindClusters, with a resolution of 0.2. Cluster marker identification was conducted using the Seurat function FindAllMarkers on the RNA assay (test.use = “LR”, latent.vars = “name”, min.pct = 0.25, logfc.threshold = 0.25) and conserved markers per cluster were computed across samples. Functional enrichment analysis of upregulated markers (avg_log2FC > 0, FDR < 0.05) was performed with clusterProfiler::enrichGO (BP/MF/CC, BH correction), using org.Hs.eg.db. The identity of the cell types within each cluster was inferred based on specific marker enrichment using enrichR and the “CellMarker 2024” database (Kuleshov et al., 2016). To assess the degree of stemness, a stemness score based on the expression of *SOX2* and *NES* genes (canonical stemness markers) was computed using the Seurat AddModuleScore function, and the percentage of cells with high outlier level of this score was calculated per sample. Cluster/sample composition was visualized as both percentage and count barplots.

The trajectory inference and pseudotime analysis was performed with Monocle3 R package (Trapnell et al., 2014), for which the Seurat object was converted into a cell_data_set object; cluster IDs (seurat_clusters) and sample labels (name) were propagated to colData. To ensure consistency with the Seurat embedding, the UMAP coordinates were injected into the cds object and partitioning was disabled (learn_graph(use_partition = FALSE)). The principal graph was learned on the injected UMAP coordinates with default monocle3 settings. The root cell was explicitly defined as the point “Y_103” within the Seurat cluster “1”. The continuous pseudotime vector obtained from monocle3::pseudotime(cds) was transferred back to Seurat metadata and used to: (i) plot UMAPs colored by pseudotime globally and faceted by sample; (ii) compare cell pseudotime distributions by sample and cluster (violin + density plots); and (iii) rank genes by their association with the learned graph using monocle3::graph_test(neighbor_graph = “principal_graph”). For visualization of dynamic gene programs, the top 50 genes with the smallest q_value and highest Moran’s I statistics from monocle3 graph_test were selected. Their normalized expression values (Seurat GetAssayData(slot = “data“)) were ordered by increasing pseudotime and visualized with ComplexHeatmap, including top annotations for pseudotime (continuous) and sample, and row annotations for gene biotype (lncRNA/protein_coding) according to GENCODE v47.

### Immunofluorescence and quantitative confocal microscopy analysis

Neurospheres were mechanically dissociated and seeded as a single-cell suspension on Cultrex™-coated coverslips at a density of 2 × 10^4^ cells/cm^2^. The following day, cells were fixed with 4% paraformaldehyde for 10 min at room temperature by gently replacing the culture medium. Samples were then washed with PBS, permeabilized with 0.3% Triton X-100 in PBS for 15 min, and blocked with 3% BSA in PBS for 30 min.

Primary antibodies against Nestin (10C2 Mouse mAb, Cell Signaling Technology, cat. #33475; dilution 1:1000) and Ki67 (Rabbit mAb, Abcam, cat. #ab16667; dilution 1:500) were incubated in 3% BSA and 0.3% Triton X-100 in PBS for 24 h at 4 °C. After primary antibody incubation, samples were washed multiple times with 3% BSA in PBS and then incubated with secondary antibodies—Donkey anti-Rabbit IgG (H+L) Highly Cross-Adsorbed Secondary Antibody, Alexa Fluor™ Plus 488 (Invitrogen, cat. #A32790TR; dilution 1:1000), Donkey anti-Mouse IgG (H+L) Highly Cross-Adsorbed Secondary Antibody, Alexa Fluor™ Plus 555 (Invitrogen, cat. #A32773; dilution 1:1000), and Alexa Fluor™ Plus 750 Phalloidin (Invitrogen, cat. #A30105)—for 1 h at room temperature in 3% BSA in PBS. Finally, samples were extensively washed and mounted with Vectashield antifade mounting medium (Vector Laboratories, cat. #H-1000-10).

All images were acquired in sequential scanning mode. XYZ image stacks were acquired using a ×20 objective with a raster size of 1024 × 1024 pixels in the X–Y plane. Imaging was performed using a Leica Stellaris 8 confocal microscope. Three-dimensional (3D) reconstructions were generated using Leica Application Suite X (LAS X). Image visualization and quantitative analyses were performed using LAS X and FIJI software.

### Statistical analysis

Statistical analysis was performed using GraphPad Prism 9 software (GraphPad Software). Data are presented as mean ± SEM. Statistical significance was assessed using an unpaired Student’s t-test or two-way ANOVA followed by Tukey’s multiple-comparisons test, as appropriate. A p value < 0.05 was considered statistically significant.

## Results

**FIGURE 1:**
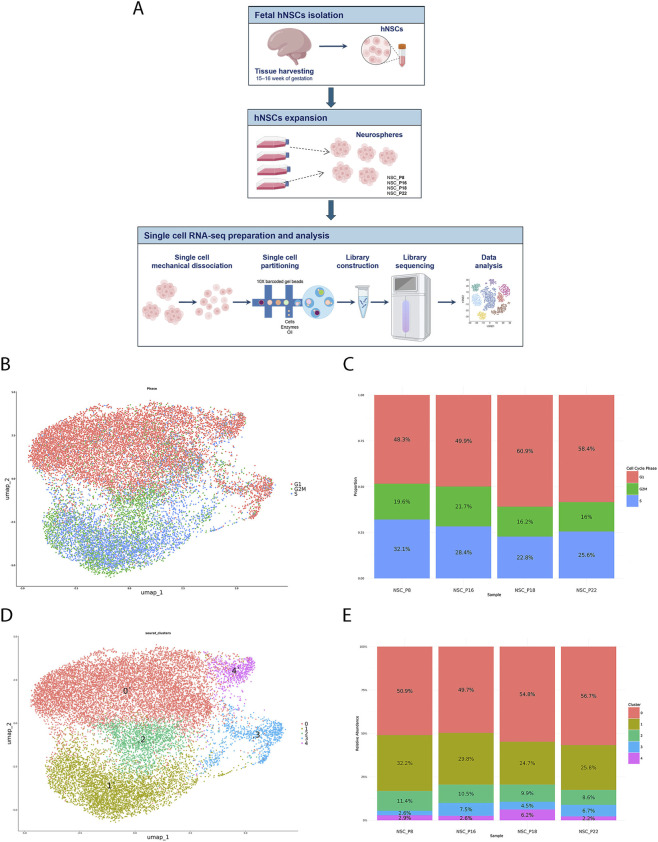
Single-cell transcriptome profiling analysis of fetal hNSCs across cell culture passages. **(A)** Schematic representation of the experimental workflow. Human fetal neural stem cells were isolated from fetal brain tissue, expanded *in vitro* as neurospheres and processed for single-cell RNA sequencing using the 10X Genomics platform. **(B)** UMAP plot showing the clustering of cells after integration of all samples, using Harmony batch correction. Each point represents a single cell, colored by cluster identity. **(C)** Bar plot showing the relative proportion of cells from each sample within each cluster, highlighting differences in representation across passages. **(D)** UMAP visualization of the same integrated dataset, with cells colored by sample of origin to illustrate the distribution of the four neural stem cell passages across clusters. **(E)** Bar plot summarizing the composition of each passage in terms of cluster proportions, showing the progressive shift in cluster distribution with increasing passage number.

To investigate the cell identity of the hNSCs purified from developing SVZ and to provide an accurate characterization of their cellular states and differentiation trajectories, we performed single cell RNA sequencing (scRNA-seq), using the 10X Genomics technology, on four hNSC samples, isolated from developing brain tissue of fetuses that died for spontaneous miscarriages at 15–16 weeks of gestation (GW), using a well-established procedure (Profico et al., 2022) (Figure 1A). To dissect and characterize both intra- and inter-sample transcriptomic variability across culture passages, we analyzed four *in vitro* culture passages (P): P8, P16, P18, P22. In total, we initially identified 21,161 cells (P8: 3,390 cells; P16: 4,464 cells; P18: 8,624 cells; P22: 4,683 cells), with a median of 5,282 UMI counts per cell and a median of 2,326 detected genes per cell (Supplementary Table S1). After normalization and rigorous quality control (Supplementary Figures S1A,B), we excluded cells expressing fewer than 500 genes or exhibiting high mitochondrial RNA content (>20%) or elevated *MALAT1* expression (>5%), which are typically indicative of stressed or non-viable cells (Supplementary Figures S2A–C). Following this filtering step, 17,096 total cells remained. Principal component analysis (PCA) revealed that the major source of variance was linked to cell-cycle phase (Supplementary Figure S3). Dimensionality reduction with UMAP and graph-based clustering, at a resolution of 0.2, identified five distinct subpopulations. Using Seurat’s *CellCycleScoring* function with the canonical marker gene sets, we determined the cell cycle phase of each cell. As expected for a stem cell population, UMAP visualization revealed a clear separation between cells in G1 phase and those in active proliferation (S or G2/M) (Figure 1B). No separation was observed between cells in S and in G2/M phases, as, to minimize the impact of cell cycle heterogeneity, the difference between the G2/M and S phase scores was regressed out. Interestingly, the proportion of cells in each cell-cycle phase did not differ markedly across passages, though a slight increase in G1 phase cells was observed in later passages (P18 and P22) (Figure 1C). The post-clustering UMAP (Figure 1D) revealed five well-defined clusters, with some (e.g., cluster 1) compact and others interconnected (e.g., cluster 2), thus suggesting a continuous of transcriptional states. This pattern was consistent with the biology of NSCs, which span a gradient of stemness, proliferation, and early differentiation. Highly similar results were obtained using tSNE-based dimensionality reduction (Supplementary Figures S4A,B), confirming the robustness of our analysis. Comparison of cluster proportions across the earliest (P8, P16) and the latest (P18, P22) passages (Figure 1E) revealed a slight increase in G1 phase relative to pre-differentiating (cluster 0) compared to proliferating cells (cluster 1).

**FIGURE 2:**
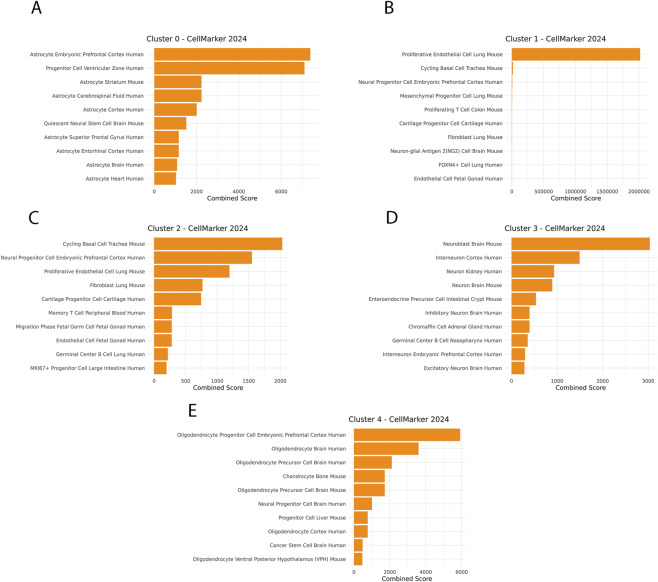
Fetal hNSCs are mainly composed of cycling cells with subpopulations committed to pre-differentiation. The five clusters were analyzed by the “CellMarker 2024 database,” testing for significant enrichment of gene sets. Data are shown as bar plots ranked by combined score. **(A)** Cluster 0 shows enrichment for astrocytic and quiescent neural stem cell signatures, suggesting a stem-like and early progenitor identity. **(B)** Cluster 1 displays enrichment for endothelial and mesenchymal-like profiles, possibly reflecting contaminant or niche-associated cells. **(C)** Cluster 2 is enriched in proliferative and neural progenitor markers, consistent with actively cycling neural stem cells. **(D)** Cluster 3 shows high enrichment for neuronal precursor and interneuron markers, indicating early differentiation. **(E)** Cluster 4 is enriched for oligodendrocyte progenitor and chondrocyte-related markers, suggesting glial-biased differentiation potential.

To identify cluster-specific marker genes, we applied the Seurat *FindAllMarkers* function to detect genes upregulated in each cluster relative to all others and then retained only markers conserved across all samples (Supplementary Table S2). Cluster annotation, to assign relevant cell identities, was carried out using the *enrichR R* package with the “CellMarker 2024” database (Kuleshov et al., 2016) (Supplementary Table S3). This analysis showed cluster 0 with an enrichment for astrocytic and quiescent neural stem cell signatures, suggesting a stem-like and early progenitor identity (Figure 2A); cluster 1 as composed by actively proliferating cells (Figure 2B), cluster 2 as composed by a transitional population (Figure 2C), clusters 3 as composed by neuronal progenitors (Figure 2D) and cluster 4 as composed by oligodendrocyte progenitors (Figure 2E).

**FIGURE 3:**
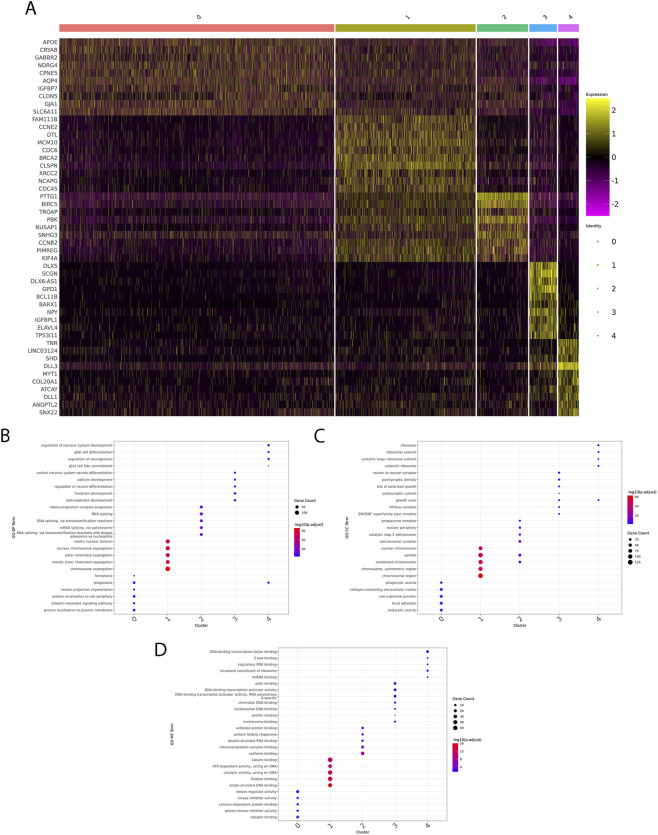
Heterogeneity in the cellular composition of fetal hNSCs reflects changes in gene expression signatures. **(A)** Heatmap showing the top 10 cluster-specific marker genes identified by differential expression analysis. Each column represents a single cell, and each row a gene, scaled by z-score. Distinct transcriptional signatures characterize the five neural stem cell clusters, including proliferative markers, differentiation-associated genes, and lncRNAs. **(B–D)** Gene Ontology (GO) enrichment analysis of cluster markers. The top enriched biological processes, cellular components and molecular functions are displayed for representative clusters.

In agreement with results of both analyses of the most variable features, on which the PCA was calculated (Supplementary Figure S5) and of the discovery of marker genes, the main drivers of cluster formation were: *APOE* and *AQP4* (cluster 0), histone and mitotic-spindle genes, linked to the S-phase (cluster 1), antisense RNA *DLX6-AS1* (cluster 3), and *DLL3* (cluster 4) (Figure 3A). Interestingly, *APOE* and *AQP4* are considered gene markers of NSC quiescence (Urbán et al., 2019), thus suggesting that cluster 0 could be composed by stem cells being at different depths of quiescence, forming a continuum of developmental stages along the quiescence to a start of activity trajectory. To gain functional insights into the biological processes underlying each transcriptionally defined cell cluster, we performed Gene Ontology (GO) enrichment on the cluster markers, summarized across the three GO domains: Biological Process (BP), Cellular Component (CC), and Molecular Function (MF). In the Biological Process category cluster-specific enrichments reflected distinct cellular programs (Figure 3B). Cluster 0 was enriched in terms related to protein localization and signalling, including protein localization to the cell periphery and integrin-mediated signalling pathway. Cluster 1 showed strong enrichment in processes associated with cell division and chromosome dynamics, such as mitotic nuclear division, chromosome segregation, and sister chromatid segregation. Cluster 2 was dominated by terms related to RNA processing and splicing (e.g., mRNA splicing via spliceosome), while clusters 3 and 4 were enriched in neurodevelopmental processes, including regulation of neuron differentiation, pallium and forebrain development. In the Cellular Component category, a similar functional stratification was observed (Figure 3C). Cluster 0 was associated with extracellular and adhesion structures, such as collagen-containing extracellular matrix, focal adhesion, and cell-substrate junction. Cluster 1 again showed strong enrichment for chromosomal structures, including condensed chromosome and centromeric region, consistent with its proliferative profile. Cluster 2 was enriched for RNA- and protein-processing complexes, such as spliceosomal complex, proteasome complex, and SWI/SNF chromatin remodelling complex. Cluster 3 was linked to neuronal compartments, including postsynaptic density and growth cone, while cluster 4 was enriched in ribosomal components, such as cytosolic ribosome and ribosomal subunit. In the Molecular Function category (Figure 3D), cluster 0 was enriched for extracellular binding and signalling functions, including integrin binding and protein kinase inhibitor activity. Cluster 1 displayed enrichment in DNA- and chromatin-binding activities, such as DNA-binding transcription factor binding, nucleosome binding, and histone binding. Cluster 2 was enriched in RNA- and protein-related functions, including RNA binding and protein folding chaperone activity. Cluster 3 was associated with regulatory activities on nucleic acids and transcription, including DNA-binding transcription activator activity and chromatin DNA binding. Finally, cluster 4 showed strong enrichment in ribosome-related functions, such as structural constituent of ribosome and miRNA binding. Overall, GO results indicated that each cluster was characterized by highly specific functional programs, ranging from cell cycle and mitotic activity (cluster 1) to RNA processing and chromatin remodelling (cluster 2), to neurodevelopmental and neuronal functions (clusters 3 and 4), while cluster 0 is primarily linked to extracellular signalling and adhesion processes.

**FIGURE 4:**
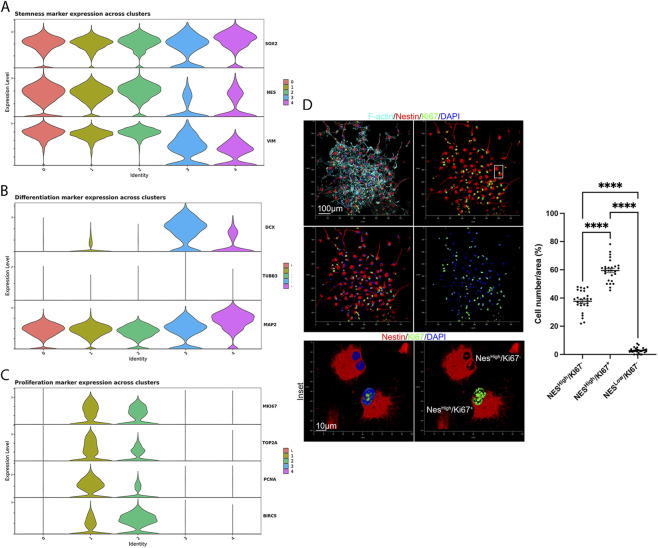
Cluster-specific expression patterns of stemness, proliferative, and differentiation markers in fetal hNSCs. **(A)** Violin plots showing the expression levels of canonical stemness markers across the five neural stem cell clusters. High expression in clusters 0–2 confirms their undifferentiated and self-renewing identity. **(B)** Violin plots of neuronal differentiation markers highlighting increased expression in clusters 3–4, consistent with early neuronal commitment. **(C)** Expression patterns of proliferation markers showing strong enrichment in clusters 1–2, indicative of actively cycling neural stem cells. **(D)** Quantitative immunofluorescence analysis of Nestin (red) and Ki67 (green). Cytoskeletal F-actin was visualized with phalloidin (cyan), and nuclei were counterstained with DAPI (blue). Two major populations were identified: Nestin^high^/Ki^67−^ cells (Cluster 0) and Nestin^high^/Ki^67+^ cells (Clusters 1 and 2). A minor Nestin^low^/Ki^67−^ population likely corresponds to Clusters 3 and 4. Representative images. Each point represents the percentage of cells per randomly selected field (n = 3 per group).

We next examined the expression of key marker genes for: stemness (*SOX2*, *NES*, *VIM)* (Figure 4A); cell differentiation (*DCX, TUBB3*, *MAP2)* (Figure 4B); cell proliferation (*MKI67, TOP2A, PCNA, BIRC5)* (Figure 4C). We observed that *SOX2*, *NES* and *VIM* were broadly expressed, though *NES* decreased only in clusters 3 and 4. Interestingly, clusters 3 and 4 displayed a bimodal distribution of stemness marker expression, suggesting a mixed population of undifferentiated and pre-differentiating cells. Indeed, cluster 3 and 4 also showed an upregulation of pre-differentiation and lineage-commitment markers (*DCX* and *MAP2)*. Clusters 1 and 2 exhibited the highest expression of proliferation markers, consistent with cell-cycle analysis (Figure 1D). Of note, we observed that cluster 0 did not display any expression of proliferation markers but, unlike cluster 3 and 4, it showed a high level of expression of *NES*, thus still supporting that this cluster could be composed of quiescent stem cells. To assess changes in pluripotency over successive passages, we computed a stemness score for each cell, based on the combined expression of *SOX2* and *NES* (Supplementary Figure S6). Across passages, the proportion of low-scoring (stemness-losing) cells slightly increased, whereas the majority of cells retained high expression of stemness markers regardless of passage (Supplementary Figure S7). We also performed quantitative confocal microscopy on hNSCs, categorizing cells based on Nestin and Ki67 expression. We identified two predominant populations: Nestin^high^/Ki^67−^ (approx. 36%) and Nestin^high^/Ki^67+^ (approx. 60%) (Figure 4D). This result was consistent with our scRNA-seq data; specifically, Nestin^high^/Ki^67−^ cells likely correspond to Cluster 0, while Nestin^high^/Ki^67+^ cells correspond to Clusters 1 and 2. A minor population of Nestin^low^/Ki^67−^ cells (3%) was also detected, potentially representing Clusters 3 and 4.

**FIGURE 5:**
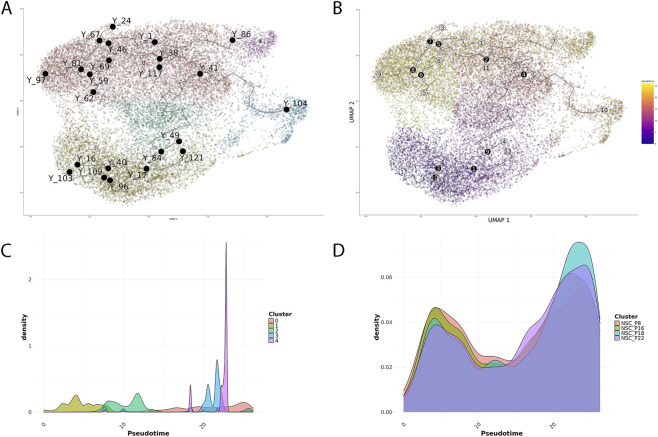
Trajectory inference highlights a gradual transition of fetal hNSCs along pseudotime. **(A)** UMAP representation of the integrated dataset colored by cluster, with pseudotime trajectory. Black dots and connecting lines represent the inferred developmental path and branch points. The trajectory starts from proliferative neural stem cell populations and extends toward more differentiated states. **(B)** UMAP colored by pseudotime values, showing a gradual and continuous transition from early to late pseudotime along the main axis of variation. **(C)** Distribution of pseudotime values across clusters, illustrating the relative positioning of each cell population along the developmental trajectory. Early pseudotime values correspond mainly to proliferative clusters, while late pseudotime regions include differentiating progenitors. **(D)** Density distribution of pseudotime for the four passages (P8, P16, P18, P22), highlighting a progressive enrichment of late pseudotime cells with increasing passage number, consistent with reduced stemness and increased differentiation potential.

Integration of differential expression and gene ontology analysis delineated a continuum of states, from proliferative neural stem/progenitor populations enriched in histone and replication-associated genes, through transitional clusters balancing proliferation and lineage priming, to transcriptionally committed neuronal and glial lineages. To capture this continuum quantitatively, we applied trajectory inference and pseudotime analysis using Monocle3 (Trapnell et al., 2014). Cells were ordered along pseudotime, revealing 10 branch points and 12 terminal states (Figure 5A). Several termini (e.g., 7, 9, and 10) traced clear trajectories toward glial precursors and neurons (Figure 5B). Branch point 4 marked NSC commitment toward neuronal (cluster 3) and glial lineages (cluster 4). Early pseudotime segments reflected a self-renewing NSC pool maintaining stemness, while seeding downstream lineages. Cell-density distributions along pseudotime supported a progressive continuum (Figures 5C,D): cluster 1 dominated early pseudotime (≈0–6), cluster 2 peaked mid-trajectory (≈8–13), cluster 3 spanned transitional regions (≈18–21), and clusters 4 and 0 marked terminal neuronal and glial states (≈20–22). Overall, the largely sequential, only partially overlapping distributions supported a continuous progression from a stem-like pool through intermediate states to two terminal lineages, mirroring the branch points and termini inferred from pseudotime and lineage analyses. When stratified by cell culture passage, the distributions remained broadly overlapping (each passage contains a continuum of states), yet a clear trend emerged. The early passages (P8 and P16) were relatively enriched at early pseudotime, whereas the late passages (P22 and especially P18) shifted toward late pseudotime, with NSC_P18 showing the most pronounced terminal enrichment (Figure 5D; Supplementary Figure S8).

**FIGURE 6:**
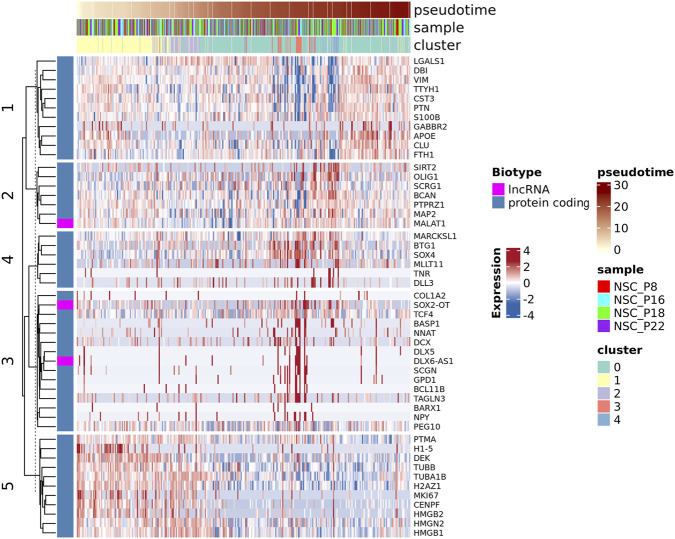
Gene expression dynamics across pseudotime reveal coordinated activation and repression of stemness and differentiation programs. Heatmap showing the expression profiles of significantly regulated genes ordered along pseudotime. Each column represents a single cell and each row a gene, scaled by z-score. The top annotations indicate pseudotime progression, sample of origin, and cluster identity. Genes are clustered by expression patterns reflecting transitions from proliferative/stemness states to neuronal differentiation. Both protein-coding genes and lncRNAs (highlighted in magenta) are shown, illustrating coordinated activation and repression of transcriptional programs driving neural stem cell maturation.

Finally, Monocle3’s graph_test() identified genes varying coherently along pseudotime (Supplementary Table S4). The signature of differentially expressed genes along the obtained trajectories revealed distinct co-regulation modules that swept from early to late pseudotime (Figure 6). Early module comprised proliferative genes (e.g., *MKI67*, *CENPF*, *PTMA*, *H1-5*, *H2AZ1*, *DEK*, *HMGN2*/*HMGB1/2*), consistent with a stem-like, cycling compartment, followed by regulatory intermediates (e.g., *SOX4*, *TCF4*, *BTG1*, *NNAT*), with terminal modules marking neuronal (e.g., *DCX, DLX5, DLX6-AS1, SCGN, BCL11B, TAGLN3, NPY, MAP2, TUBA1B/TUBB*) and glial/OPC–astrocytic (e.g., *OLIG1, SIRT2, BCAN, PTPRZ1, TNR; S100B, APOE, CLU, LGALS1, COL1A2*) fates. Notably, several lncRNAs (e.g., *SOX2-OT, DLX6-AS1*) co-varied with lineage-specific modules suggesting lineage-coupled regulation. Overall, the graph-autocorrelation analysis confirmed a continuous, biologically coherent progression from proliferative/stem-like cells to lineage-committed neuronal and glial precursors, consistent with the clustering-based observations.

## Discussion

NSCs are emerging as a promising tool for the treatment of neurodegenerative diseases. Several preclinical and clinical trials have demonstrated their ability to counteract the pathological processes underlying many of these disorders. Thanks to their capacity to self-renewal and differentiate into distinct mature cell types, NSCs can integrate into nervous tissue, replace lost or damaged cells and restore neural functionality (Grochowski et al., 2018; Rahimi Darehbagh et al., 2024). Although the precise mechanisms underlying the therapeutic effects of stem cells in neurodegenerative diseases remain unclear, they likely depend on both the specific neurological condition and the stem cell type employed.

The transcriptional complexity of the developing human neocortex is still not fully understood and remains elusive. This lack of knowledge represents a major obstacle to comprehending the mechanisms governing human neurogenesis and limits the potential of hNSCs in regenerative medicine. Fetal hNSCs represent the most physiologically relevant model of early neurogenesis, showing balanced self-renewal and lineage priming and, therefore, constitute a powerful tool for NSC-based therapies targeting neurodegenerative disorders. In contrast, induced pluripotent stem cell-derived human neural stem/progenitor cells (iPSC-hNSCs), although easier to produce for therapeutic purposes, fail to fully recapitulate the physiological cellular composition and functional properties of SVZ-derived hNSCs. To gain new insights into the cellular composition of human SVZ-hNSCs derived from the developing neocortex, we performed single-cell RNA sequencing (scRNA-seq), using the 10X Genomics technology, on hNSCs isolated from the SVZ of fetuses who died from spontaneous miscarriages at 15–16 weeks of gestation. These hNSCs were obtained from a well-established and reproducible production process (Profico et al., 2022), and have previously been used in phase I transplantation trials in ALS and MS patients (Mazzini et al., 2019; Leone et al., 2023). Our clustering analysis revealed five main cellular subpopulations. The observed molecular signature of the most abundant cluster (cluster 0), such as the high expression of *AQP4* and *APOE* genes*,* that are considered stem-related quiescence gene markers (Urbán et al., 2019; Apostolou and Donega, 2025) (Figure 3) and the downregulation of genes associated with cell proliferation (*MKI67, TOP2A, PCNA, BIRC5*) (Figures 4, 6), suggested that this cluster is a population, composed by both glial progenitor and quiescent stem cells. However, the absence of a well-defined G0-specific gene set that can clearly distinguish quiescent from G1 cells, in the Seurat database used for cell cycle phase definition, currently makes it challenging to draw a more precise definition of the biological nature of this cluster. Quiescent stem cells are the principal source for maintaining stemness (Urbán et al., 2019) and their probable presence in cluster 0, that is the most abundant cluster, may underline that the isolation and expansion procedures of hNSCs is able to preserve the central stem cell pool of both the fetal and adult neurogenesis. This feature could be of great importance for the potential of these cells in the treatment of neurodegenerative diseases, as they may contain a cell pool capable of preserving themselves over time and reacting to local stimuli, actively contributing to the damaged tissue repair. Cluster 1 and 2 displayed a clear proliferative signature, characterized by strong upregulation of genes involved in DNA replication and chromatin remodelling. Within cluster 1, we also detected and quantified multiple histone transcripts. Although canonical histone mRNAs are typically non-polyadenylated and, therefore, should not be captured by standard 10X Chromium single-cell RNA-seq protocol, their enrichment can likely be explained by unspecific binding of these abundant transcripts to the poly(dT) primer of the 10X Gel beads during cDNA synthesis. The remaining clusters branched from the neural progenitor cell cluster along two principal trajectories, one following glial lineage, further subdividing into astrocyte- and oligodendrocyte-committed cells, and the other following a neuronal trajectory. Notably, among the genes enriched in the neuronal branch, we observed a strong expression of the long non-coding RNA *DLX6-AS1* (also known as EVF2), which plays a key regulatory role in neural stem cell function and forebrain development by modulating the expression of *DLX* family genes essential for GABAergic interneuron differentiation (Li et al., 2025). Beyond its specific involvement in embryonic brain development, *DLX6-AS1* exemplifies the growing importance of long non-coding RNAs (lncRNAs) in orchestrating higher-order transcriptional programs that shape the structural and functional complexity of the human brain. Increasing evidence suggests that lncRNAs contribute to species-specific regulatory layers in cortical development, influencing neuronal commitment, differentiation timing, and regional identity (Mattick et al., 2023). Although trajectory inference was inherently limited by the relatively small size and homogeneity of the analyzed cell population, it nevertheless revealed a continuum of transcriptional states reflecting progressive stages of neural stem cell maturation. Notably, the genes dynamically and significantly regulated along the pseudotime largely overlapped with those identified through the cluster-based approach, supporting both the robustness of the inferred lineage relationships and the consistency between discrete and continuous models of hNSCs progression. We also observed that, as the number of culture passages increased (P18 and P22), the proportion of cells within differentiating clusters gradually rose compared to the more stem-like clusters, which nevertheless remained abundant. To date, only a few studies have explored the single-cell transcriptomic landscape of human neural stem cells. To our knowledge, only two have focused on hNSCs derived from developing cortices: one profiling all embryonic brain cells (Fan et al., 2020) and the other, using cell-surface markers to purify NSCs and progenitor cells from by flow cytometry (Liu et al., 2023). Despite differences in isolation strategies and scRNA-seq library preparation methods, both studies complement our findings. Moreover, the hNSCs analyzed here were isolated from the fetal SVZ using a standardized *in vitro* isolation and expansion protocol, ensuring a more homogeneous and reproducible population. Other studies have examined NSCs derived from adult human SVZ (Baig et al., 2024) or hippocampus (Dumitru et al., 2025); while informative, these cells reflect the transcriptional landscape of adult brain and biological complexity of NSCs in the adult brain and lack the developmental plasticity of fetal hNSCs. Recently, a direct comparison of the transcriptome of fetal hNSCs and induced pluripotent stem cell-derived neural progenitors (hiPSC-NSCs), at both bulk and single cell levels has been performed, demonstrating a clear transcriptional divergence, with fetal hNSCs retaining a more stem-like profile (Luciani et al., 2024).

In conclusion, our single-cell transcriptional profiling of human fetal neural stem cells reinforces their value as a promising tool for regenerative medicine. Overall, although derived from a relatively limited number of samples, our data indicate that hNSCs preserve their stem identity even after prolonged *in vitro* culture expansion. The sustained expression of canonical stemness markers and maintenance of self-renewal capacity suggest a remarkable stability of their molecular signature across passages, enhancing their potential for regenerative and therapeutic applications.

## Data Availability

The data presented in this study have been deposited in NCBI SRA with the BioProject Accession number: PRJNA1425026.

## Supplementary material

SUPPLEMENTARY FIGURE S1 Violin plots showing Seurat quality control metrics for the four sequenced samples before **(A)** and after **(B)** the filtering, performed in the data pre-processing phase.

SUPPLEMENTARY FIGURE S2
**(A–D)** Scatter plots showing the number of genes detected per cell (nFeature_RNA), the number of UMIs per cell (nCount_RNA), and the percentage of reads per cell resulting from MALAT1 expression.

SUPPLEMENTARY FIGURE S3 PCA plot showing cells colored according to cell cycle phase and showing that the separation according to PC_1 and PC_2 is strongly driven by the cell cycle.

SUPPLEMENTARY FIGURE S4 tNSE plots comprehensive **(A)** and splitted by sample **(B)** post clustering.

SUPPLEMENTARY FIGURE S5 Distribution of variable features in the four neural stem cell samples (NSC_P8, NSC_P18, NSC_P22, NSC_P16). Red dots indicate the 2,000 most variable genes, while black dots represent non-variable genes. Selected genes associated with proliferation and differentiation are labeled.

SUPPLEMENTARY FIGURE S6 Violin plots showing the distribution of percentages of reads per cell for genes *SOX2, NES* and “Stem scores” in the 4 NSC samples.

SUPPLEMENTARY FIGURE S7 Relative proportion of proliferative versus non-proliferative cells across passages, showing a progressive reduction in the cycling fraction with increasing passage number.

SUPPLEMENTARY FIGURE S8 Violin plots showing the distribution of pseudotimes in the 4 NSC samples.

SUPPLEMENTARY TABLE S1 sc-RNA-seq metrics.

SUPPLEMENTARY TABLE S2 NSC clusters’ gene markers.

SUPPLEMENTARY TABLE S3 Cell type enrichment analysis of cluster-specific gene signatures using Enrichr CellMarker database.

SUPPLEMENTARY TABLE S4 principal_graph_pseudo_markers.
