# Supplementary figures and images for: Single-cell transcriptomic profiling of human fetal neural stem cells isolated from the subventricular zone
Source: Front Cell Dev Biol. 2026 Mar 10;14:1740851. doi: 10.3389/fcell.2026.1740851 (PMC13008933; doi:10.3389/fcell.2026.1740851)

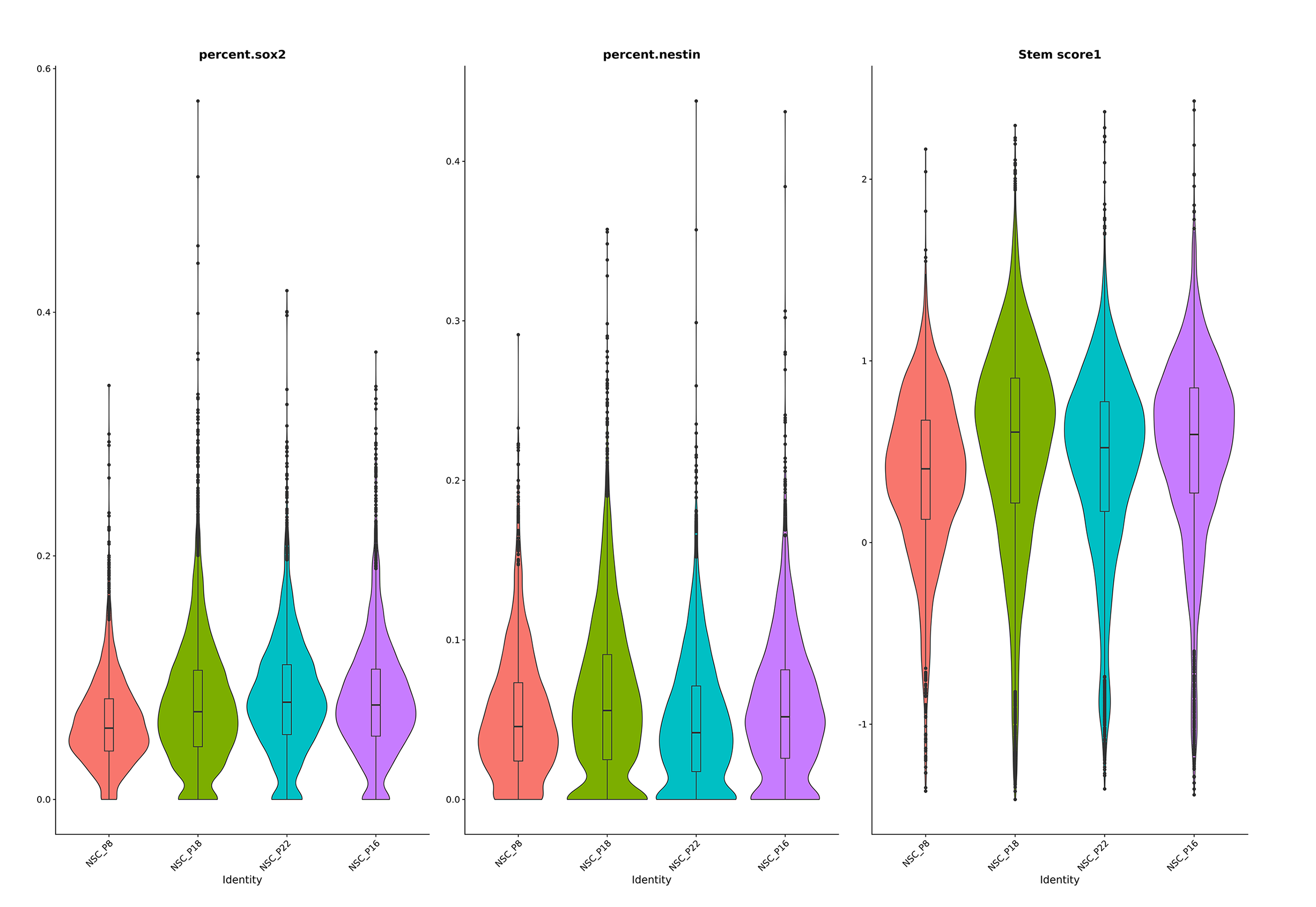

Supplement: Supplementary file 3 [file Image6.tif]

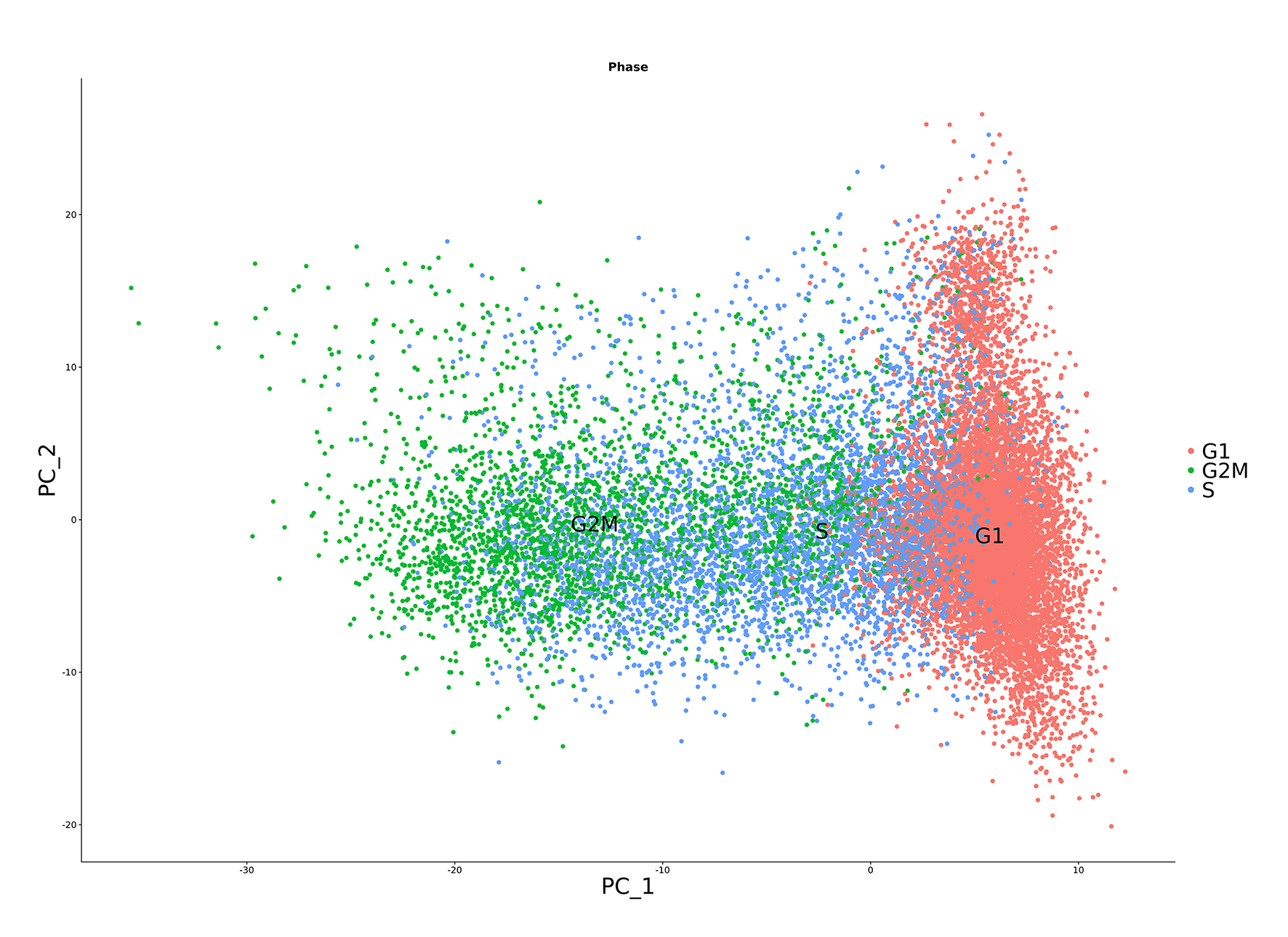

Supplement: Supplementary file 4 [file Image3.tif]

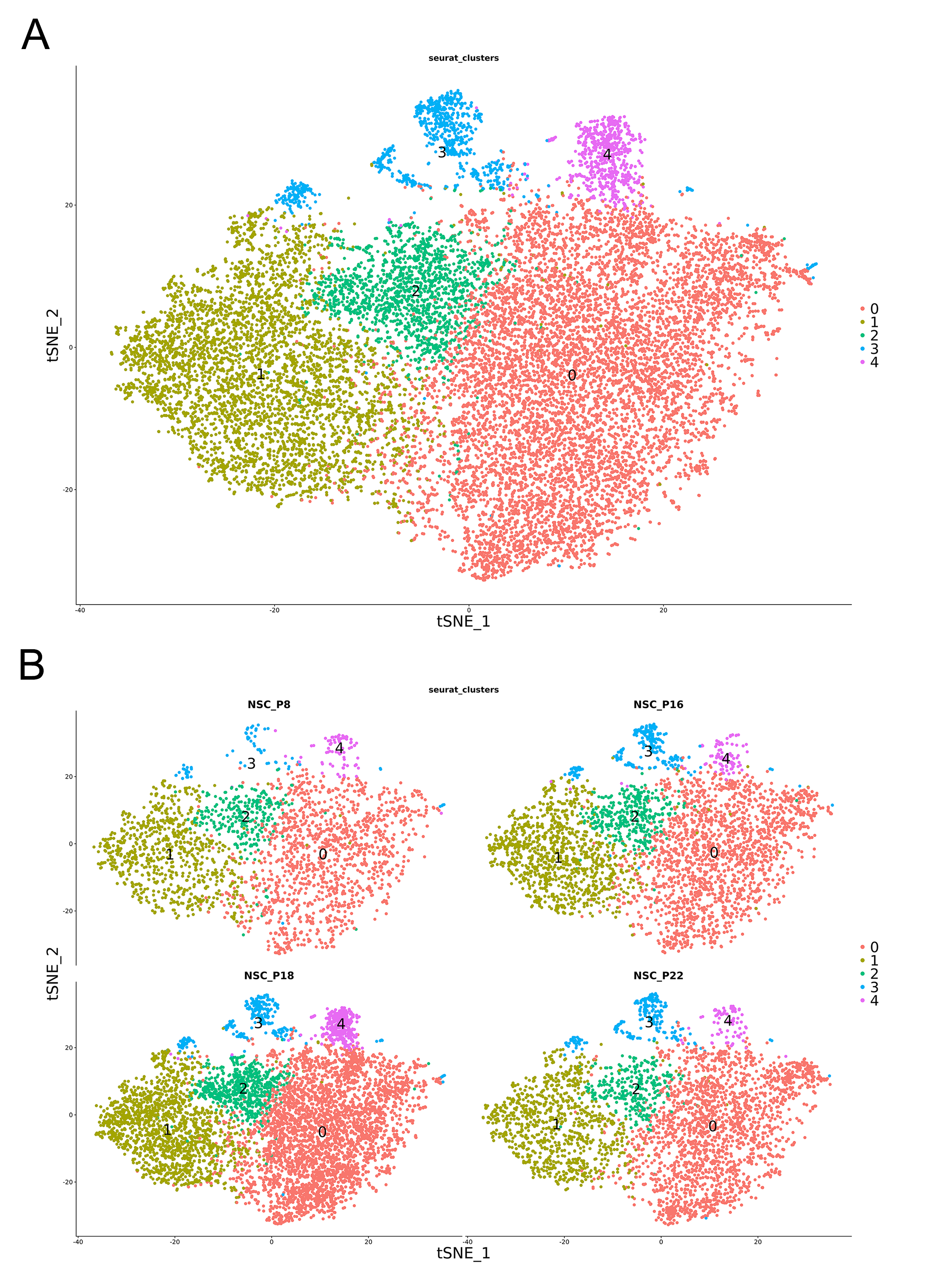

Supplement: Supplementary file 5 [file Image4.tif]

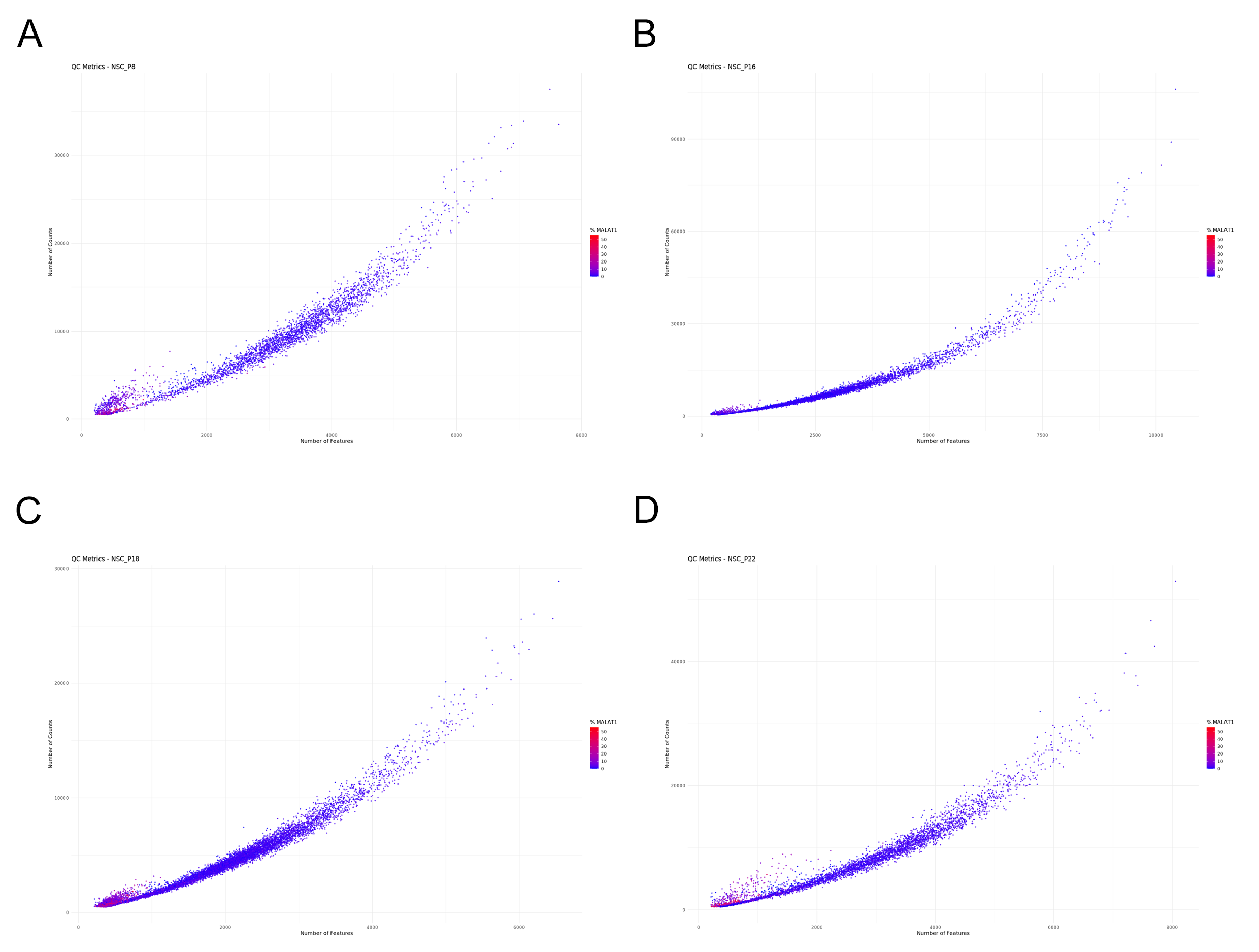

Supplement: Supplementary file 6 [file Image2.tif]

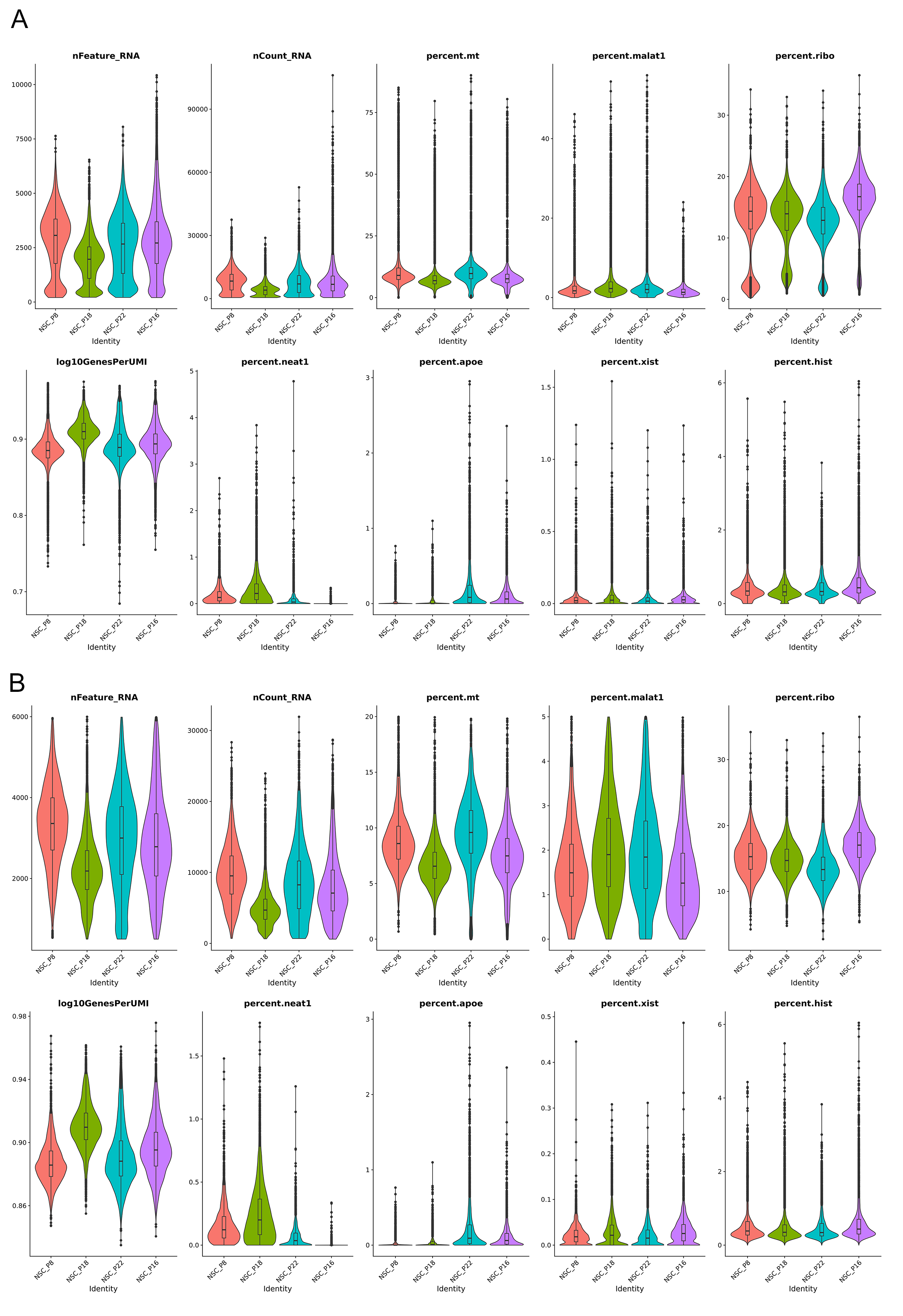

Supplement: Supplementary file 7 [file Image1.tif]

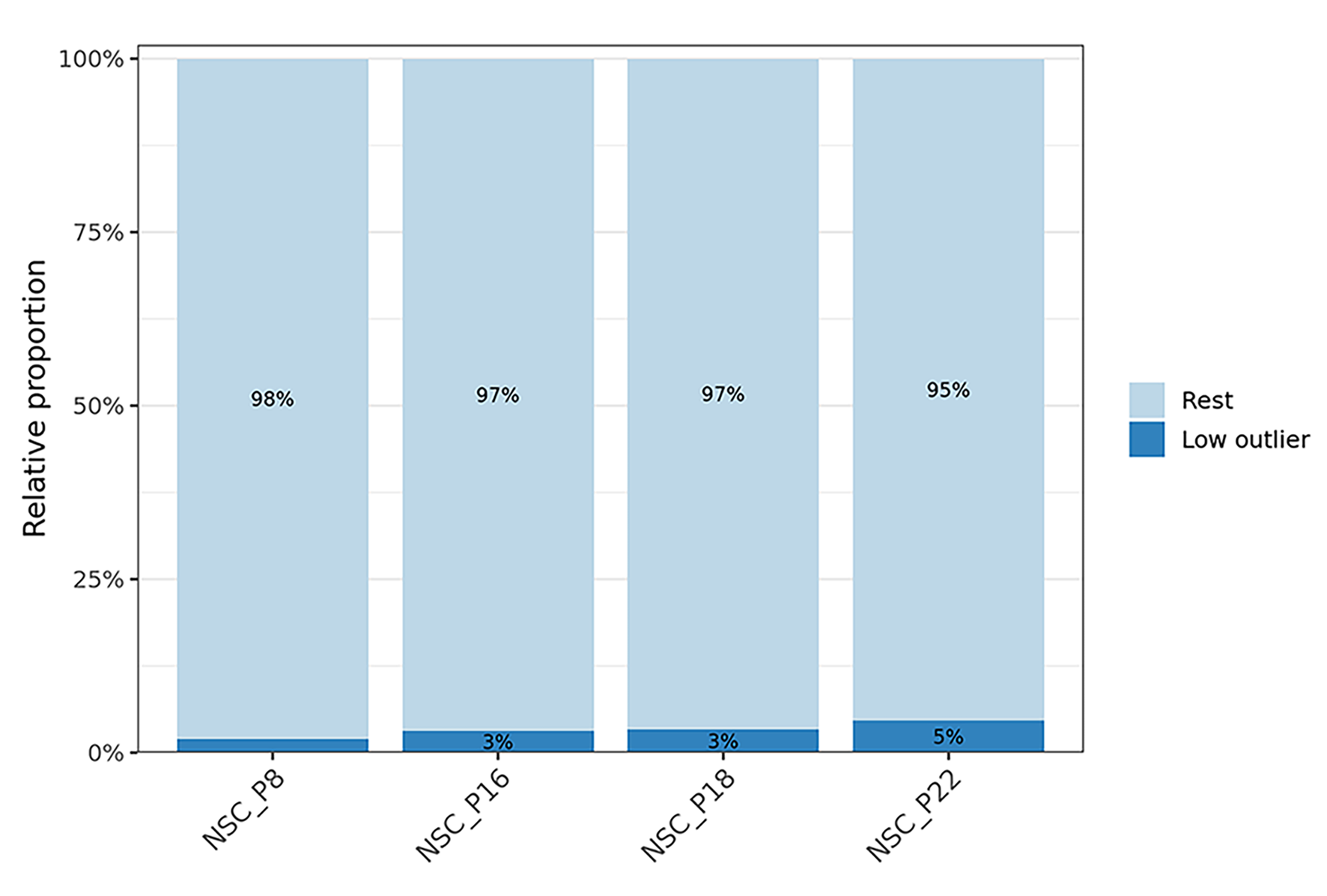

Supplement: Supplementary file 8 [file Image7.tif]

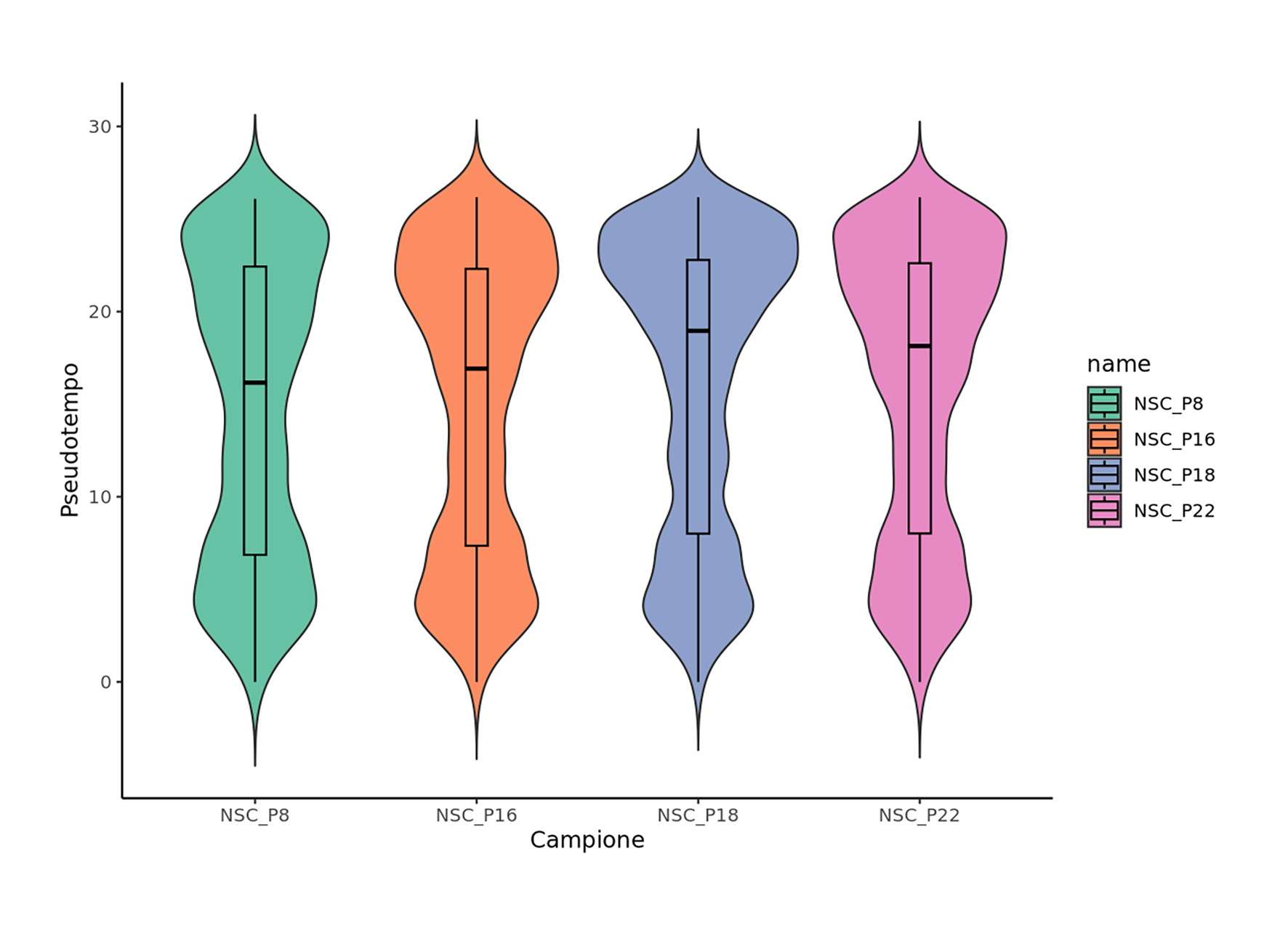

Supplement: Supplementary file 11 [file Image8.tif]

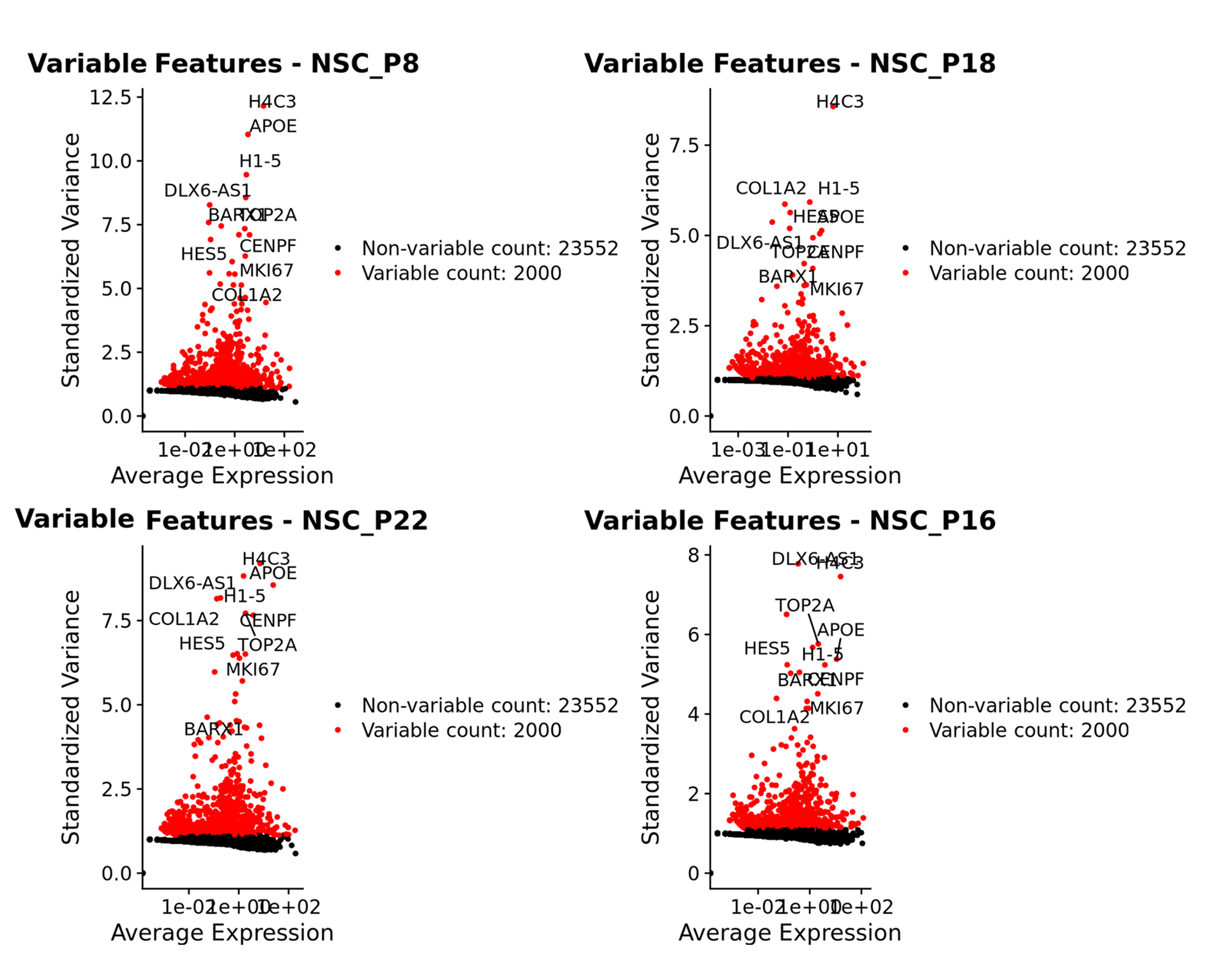

Supplement: Supplementary file 12 [file Image5.tif]
